# Online prescriptions of pharmaceuticals: Where is the evidence for harm or for benefit? A call for papers - and for reflection

**DOI:** 10.2196/jmir.3.1.e1

**Published:** 2001-01-31

**Authors:** G Eysenbach

This issue of JMIR features a provocative study which will almost certainly lead to great controversies: A "cyberdoctor" who prescribed sildenafil online compared the collected data and outcomes of his online patients with those from a traditional clinic [1]. His conclusions, based on more than 2,000 online encounters: "the Internet-based prescribing physician has more, not less, clinically relevant and useful information than was typically obtained and utilized in a specific hospital clinic setting", and there is no evidence to believe that patients have been harmed.

However - to avoid any misunderstandings - it should be stressed right up front that the study is subject to considerable limitations, as no prospective and active follow up of the "clients" could be performed, and the outcomes of the patients who didn't volunteer any follow-up information are unknown. Larger prospective studies with a more rigorous design, such as cohort studies, are urgently needed. Still, we believe that the study is groundbreaking, in a sense that this is the first study providing any sort of data about online prescribing.

In many areas of the world, online prescribing of drugs without a prior personal doctor-patient relationship is still considered unethical or even unlawful. The accompanying article by J. Henney outlines the current situation and debate in the US. The FDA is "concerned about the proliferation of sites that substitute a simple online questionnaire for a face-to-face examination and patient supervision by a health care practitioner" and believes that "the risk of negative outcomes such as harmful drug interactions, contraindications, allergic reactions or improper dosing is greatly magnified."

However, ethics and law-making should be based on evidence, just as medical practice itself is; some may argue that the current paradigm and restrictive legislation is not evidence-based, but overly paternalistic and an anachronism.

Paternalism (alluding to a child-parent relationship) has been defined as (1) being primarily intended to benefit the recipient, and (2) the recipient's consent or dissent is not a relevant consideration for the initiator [2]. To justify paternalistic medical practice and legislation, which affects to a certain degree patients' autonomy and their right to decide, we have to ask ourselves whether these actions really benefit the recipient, and whether - in the age of openness and free information - the free will of patients (to make an informed choice not to see a doctor in person but to order pharmaceuticals after an online assessment) is something which can be easily ignored.

This is bottom line: Currently, we simply do not have sufficient evidence whether, and under which conditions, online prescribing of relatively safe drugs such as the impotence drug Viagra (sildenafil citrate) actually creates more harm than benefit, or vice versa. More research is urgently needed to address questions such as which drugs can be prescribed safely and to which kinds of patients, and which safeguards we can install to monitor adverse events.

## The FDA evidence

FDA evidence for the alleged risks of online prescribing to date merely consists of a few anecdotal cases. The most frequently cited case is the story of a 52-year-old Illinois man with episodes of chest pain and a family history of heart disease, who died of a heart attack in March 1999 after buying Viagra (sildenafil citrate) from an online source that required only a completed questionnaire to qualify for the prescription. Though there is no proof linking the man's death to the drug, FDA officials say that a traditional doctor-patient relationship, along with a physical examination, may have uncovered any health problems such as heart disease and could have ensured that proper treatments were prescribed. However, it should be noted that there have been several similar cases where patients with a comparable history have died while taking Viagra, despite receiving their prescriptions at the doctor's office.

This scarcity of reports of adverse events is surprising, given that millions of pills are prescribed on the Web each year. Leading online pharmacies report that they issue more than 1,000 prescriptions a day. It has been estimated that Viagra is advertised on 4,500-15,000 Web pages, with an unknown number of distinct companies behind these pages (the affiliate programs sponsored by Viagra purveyors provide a financial incentive to Web sites which advertise their services). A very conservative estimate would be that at least 150 distinct companies exist on the Web which prescribe Viagra every day [3].

## Studies evaluating cyberpharmacies from the outside

A number of studies have shown that prescription drugs are easily available online. The bad reputation of online pharmacies may also come from research evidence which suggests that many sites selling prescription drugs supply consumers with drugs when, for medical reasons, they shouldn't have.

At least two studies [3,4] attempted to determine the quality of the service provided by Internet consulting physicians by actually ordering drugs through these services. The two studies looked whether drugs are supplied to people for whom they are not suitable. They did so by posing as a patient with contraindications; for example, by posing as a 69-year-old female with coronary artery disease [3], or a 45-year-old male with a history of heart attack and currently taking nitrate [4], both ordering Viagra. Despite clear contraindications, 3 out of 10 (30%) [3] and 1 out of 5 (20%) [4] of the services actually delivered Viagra to these patients. In another scenario, the weight-loss drug Xenical was delivered in 5 out of 5 tested services to a fictitious patient with lifestyle-related obesity and a BMI of 28, which is not considered an indication for Xenical [4]. Other issues criticized by these studies include: some of the services did not gave an accurate reason for not delivering the drug; some of the services did not obtain an appropriate medical history; and some of the services used inappropriate medical terminology or used only the brand names of drugs (without generic names).

Some studies also only looked at the information offered on these sites [3,5,6]. The three studies dealing with drug information on e-commerce sites were all conducted between February and May 1999 and all focussed on sildanefil sites (one in addition looked at sites selling finasteride [5]). To evaluate the quality of these sites, authors extracted prices [3,5], checked for completeness of online-history taking and/or information provided on the site e.g. pertaining to contraindications [3,6], and the presence/absence of disclaimers and/or liability waivers [3,6].

**Figure 1:**
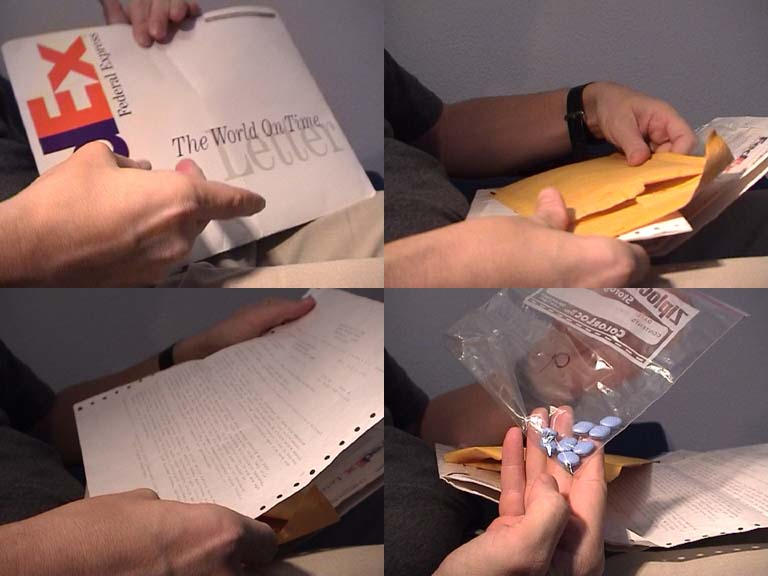
Figures 1-4, Pills by mail: Video captures () from test orders of different e-pharmacies. 3 out of 10 e-pharmacies delivered to the hypothetical obese 69-years old woman claiming to have "orgasm problems" [3]

## Quality criteria for online prescribing

In addition to some "good practice" standards for any type A online doctor-patient relationship, [3,7,8] several quality criteria for online consultations and online prescribing should be taken into account, and should potentially be a prerequisite for any certification program:

- Informed consent: Patients must be fully informed about the risks of online prescribing in general, and the risks and side-effects of the prescribed drug in particular.
- A thorough medical history should be taken, especially as cyberdoctors don't have the patient's records in front of them and must rely on the information volunteered by the patient. In one study, only 3 out of 10 online pharmacies prescribing Viagra on the Internet asked about retinitis pigmentosa as a potential contraindication, and in 8 out of 10 services the history obtained was judged inadequate [3]. In another study, an expert panel of two general practitioners (GPs) and a pharmacist judged that in one of four tested cyberdoc sites, the history taken was inadequate, and that those services prescribed a anti-hypertension drug without making sufficient steps to arrive at a diagnosis [4].
- Patient-understandable language: Advice should be provided in a patient-understandable language. One study said that in one of four tested cyberdoc sites, the answer given was "much too full of medical jargon" and "read more like an extract from a medical text rather than advice for this specific patient," and in addition used "poor English" [4]. Sites selling Viagra to consumers used, in 70% of cases, inappropriate medical terminology in the medical history forms [3]. One study also evaluated the friendliness of responses [9].
- Continuity of care: Is the information designed to support existing patient-physician relationships? What type of follow-up is offered? Is the patient's own GP informed about any treatment given or recommended on the Internet?
- Accountability: Consumers should know who is giving the advice and what that person's qualifications are. Armstrong noted that none of the 77 sites offering Viagra provided specific information about the qualifications of the physician [6]; and Bloom noted that the address of the consulting physician, and his or her specialty, location, and qualifications were given in none of the sites reviewed [5,6]. Another study said that only on 1 out of 10 visited sites selling Viagra or Xenical revealed the name or qualifications of the doctor on the Web [4]. Even when the consumer actually ordered the drug and received a "prescription" or is notified that the prescription was declined, this information is given only in a minority of the cases. In one study, only in 2 out of 10 sites which issued or declined a Viagra presciption revealed the identity of the consulting doctor [3].
- Response/delivery time. Varying response times of a few hours to several days were measured in studies [10,11] Viagra was delivered in 6, 10, and 34 days [3].
- Security and patient confidentiality: As e-pharmacies store large amounts of highly sensitive data (including the results of online-assessment forms containing personal medical data, as well as the name and address of the purchaser), security is a particular concern.

The FDA requires online pharmacies to post information on their Web sites about their ownership, state licensure, name of the pharmacist in charge, and a phone number where consumers can contact the pharmacist.

## Conclusion: From Rx and OTC (over-the-counter) to OTI (over-the-Internet) drugs?

Online prescribing of pharmaceuticals is - much as other forms of online interventions such as online psychotherapy or even educational interventions - a two sided coin. This of course is true for many (if not all) interventions in medicine - no treatment is without risks and side effects, and it is always crucial to balance potential benefits against their risks. In order to balance the risks and the benefits, we need scientific evidence for the probabilities of certain outcomes, and need to estimate the "utility" patients and society put on certain outcomes.

Online prescriptions may, under certain circumstances, be not more "potentially dangerous" than, for example, self-medication with OTC (over-the-counter) drugs (for which consumers do not need any prescription and bypass physicians completely) provided that such services are appropriately monitored, and the right drugs for this new form of prescribing are chosen.

Thus, I would argue that we should consider the introduction of a new class of drugs which we may call OTI: over-the-Internet drugs, which are safe enough to be prescribed over the Internet, but not safe enough for OTC use. They may in the future constitute a middle ground between OTC and Rx (prescription) drugs. For drugs to qualify as OTI, a preexisting patient-physician relationship and/or a thorough physical examination must not be crucial, an online assessment or email interaction may be considered sufficient, and the benefits should greatly outweigh the risks. To be able to decide which factors may make an OTI drug out of a Rx drug we - again - need appropriate studies.

## A standing call for papers and case reports

We are interested in getting feedback from professionals as well as from patients on this issue. We welcome all sorts of papers, including short letters to the editor, informed comments, or full original research studies. We are inviting a look at all aspects of this topic, including but not limited to surveys of patient preferences, case reports or controlled studies of patient outcomes, legal commentaries or cost-effectiveness studies. We would also like to hear from consumers who have had positive or negative experiences with such services, who have been harmed or benefited from online prescriptions or - more broadly - online therapy in general. We also hope to hear from physicians who have encountered patients who have been harmed or who have benefited from this practice. Finally, we would also like to hear proposals or implementations of informatics, policy solutions or other mechanisms to monitor adverse reactions, and the dissemination of the collected information.

*Gunther Eysenbach, MD*

*Editor,*

*Journal of Medical Internet Research*
